# Shortening the Current Opioid Misuse Measure via computer-based testing: a retrospective proof-of-concept study

**DOI:** 10.1186/1471-2288-13-126

**Published:** 2013-10-20

**Authors:** Matthew D Finkelman, Ronald J Kulich, Driss Zoukhri, Niels Smits, Stephen F Butler

**Affiliations:** 1 Department of Public Health and Community Service, Tufts University School of Dental Medicine, Boston, MA 02111, USA; 2 Craniofacial Pain Center, Tufts University School of Dental Medicine, Boston, MA 02111, USA; 3 Department of Diagnosis and Health Promotion, Tufts University School of Dental Medicine, Boston, MA 02111, USA; 4 Department of Clinical Psychology, VU University Amsterdam, Amsterdam, the Netherlands; 5 Inflexxion, Inc., Newton, MA 02464, USA

**Keywords:** Substance abuse, Chronic pain, Opioids, Questionnaire, Respondent burden, Computer-based testing

## Abstract

**Background:**

The Current Opioid Misuse Measure (COMM) is a self-report questionnaire designed to help identify aberrant drug-related behavior in respondents who have been prescribed opioids for chronic pain. The full-length form of the COMM consists of 17 items. Some individuals, especially compromised individuals, may be deterred from taking the full questionnaire due to its length. This study examined the use of curtailment and stochastic curtailment, two computer-based testing approaches that sequentially determine the test length for each individual, to reduce the respondent burden of the COMM without compromising sensitivity and specificity.

**Methods:**

Existing data from *n* = 415 participants, all of whom had taken the full-length COMM and had been classified via the Aberrant Drug Behavior Index (ADBI), were divided into training (*n* = 214) and test (*n* = 201) sets. Post-hoc analysis of the test set was performed to evaluate the screening results and test lengths that would have been obtained, if curtailment or stochastic curtailment had been used. Sensitivity, specificity, and average test length were calculated for each method and compared with the corresponding values of the full-length test.

**Results:**

The full-length COMM had a sensitivity of 0.703 and a specificity of 0.701 for predicting the ADBI. Curtailment reduced the average test length by 22% while maintaining the same sensitivity and specificity as the full-length COMM. Stochastic curtailment reduced the average test length by as much as 59% while always obtaining a sensitivity of at least 0.688 and a specificity of at least 0.701 for predicting the ADBI.

**Conclusions:**

Curtailment and stochastic curtailment have the potential to achieve substantial reductions in respondent burden without compromising sensitivity and specificity. The two sequential methods should be considered for future computer-based administrations of the COMM.

## Background

The continued presence of unrelieved pain as a serious public health issue has been well-documented [1-4]. Opioids have increasingly been used in recent years to address the problem, and they may be an important component of treatment for chronic pain [5]. However, some patients who receive a prescription for opioids may be prone to non-adherence or misuse behaviors, such as escalation of their opioids, visiting multiple providers, or other overt drug-seeking behaviors [6,7]. The identification of opioid misuse is thus critical in the treatment of chronic non-cancer pain [7], but many physicians lack adequate training to make such an identification [8].

To assist clinicians in recognizing aberrant drug-related behavior among respondents who have been prescribed opioids, Butler et al. [7] introduced the Current Opioid Misuse Measure (COMM), a 17-item self-report questionnaire. Because the COMM is designed to help assess whether a given respondent is currently misusing opioids, it should be distinguished from other instruments that predict future misuse [9]. Previous research has validated the COMM against the Aberrant Drug Behavior Index (ADBI), a combination measure that incorporates information from a questionnaire taken by the patient, a questionnaire taken by the treating clinician, and urine toxicology results. The COMM was found to exhibit adequate sensitivity and specificity in both its original validation study [7] and a cross-validation study using a new population of patients [9].

Although most respondents can finish the full-length COMM in a reasonable amount of time, some individuals may be unable or unwilling to complete all 17 items. Members of compromised subpopulations (e.g., those with physical ailments and those who are at a low reading level) are less likely to accept lengthy screeners [10]. The response rate [11] and the quality of an individual’s answers to a given questionnaire [12] may be improved by administering fewer items. Shortening an instrument may also lessen the degree of stress associated with taking it and decrease the likelihood that respondents will drop out midway through [13]. Finally, reducing respondent burden is especially critical for lowering drop-out rates when a questionnaire is given to patients on multiple occasions over time [13,14]—and such longitudinal tracking of patients was listed as a goal of the COMM in its original validation study [7]. These considerations demonstrate the need for a version of the COMM that lessens respondent burden while maintaining the sensitivity and specificity of the full-length instrument.

A number of recent articles have shown that modern advancements in computer-based testing can enhance the efficiency of an assessment [15-17]. In particular, computer-based forms have the potential to achieve the same levels of sensitivity and specificity as their paper-and-pencil counterparts, but by using fewer items on average [18,19]. The reason for this advantage is that computer-based forms can track a respondent’s answers as the test progresses. By performing *interim analysis* of these answers while assessment is underway, a computer program can customize the test to the individual taking it. A well-known component of this customization is the use of *sequential stopping rules* to determine the appropriate test length for a given respondent. Two such stopping rules that have been proposed for computer-based testing are the methods of curtailment and stochastic curtailment. As will be explained in the Methods section, curtailment and stochastic curtailment attempt to shorten each respondent’s questionnaire while maintaining the same test outcome (“positive” or “negative”) that would have been made if the full-length questionnaire had been used. In retrospective analyses of responses to the Medicare Health Outcomes Survey [18] and the Center for Epidemiologic Studies-Depression (CES-D) scale [19], both methods substantially reduced the average number of items administered without compromising sensitivity and specificity. However, no previous study investigated either method as a means of enhancing the efficiency of the COMM.

The purpose of this study is twofold. First, we describe how the stopping rules of curtailment and stochastic curtailment can be applied to the COMM. Second, we evaluate how successful these rules are in reducing respondent burden and maintaining sensitivity and specificity. For the latter objective, we conducted an analysis of existing data from individuals who had already been administered the full-length COMM and been classified via the ADBI; thus, the research constitutes a “proof-of-concept” study for using a computer-based COMM in the future.

The article is organized as follows. The Methods section describes how the data were collected, how the COMM and ADBI are scored, how curtailment and stochastic curtailment can be used in conjunction with the COMM, and what analyses were performed. The Results section presents sensitivity and specificity values, the average test length of each method, and other statistics. The Discussion and Conclusions sections explore the implications of the study and present ideas for future work.

## Methods

### Participants

This retrospective study used data from two sources: the initial validation study and the cross-validation study of the COMM. Details about the subjects who participated in the initial validation study are provided in Butler et al. [7]. Briefly, the sample consisted of 227 chronic non-cancer pain patients from a hospital-based pain management center in Massachusetts, a hospital-based pain management center in Pennsylvania, and a private pain management treatment center in Ohio. As reported in Butler et al. [7], the mean (SD) age of participants was 50.8 (12.4) years, the mean (SD) number of years taking opioids was 5.7 (9.2), the percentage of females was 62%, and the percentage of Caucasians was 83%. Details about the subjects who participated in the cross-validation study are provided in Butler et al. [9]. Briefly, 226 non-cancer pain patients from pain management centers in five locations (Indiana, Massachusetts, New Hampshire, Ohio, and Pennsylvania) comprised the study population. As reported in Butler et al. [9], the mean (SD) age of participants was 51.5 (13.8) years, the mean (SD) number of years taking opioids was 5.4 (5.8), the percentage of females was 48%, and the percentage of Caucasians was 87%. All subjects (from both the initial validation study and the cross-validation study) were taking opioids at the time the research was conducted. All subjects signed an informed consent form. The Human Subjects Committee of Inflexxion, Inc. approved the initial validation study; the Human Subjects Committee of the participating clinical sites approved the cross-validation study.

### Assessments used

In both the initial validation and cross-validation studies, subjects were administered the COMM questionnaire. The items making up this questionnaire are listed in Table 1. Each item refers to the stem “In the past 30 days…” and has five possible answers: “never” (scored 0), “seldom” (scored 1), “sometimes” (scored 2), “often” (scored 3), and “very often” (scored 4). A respondent’s total score is obtained by summing the individual item scores; because there are 17 COMM items, scores on the full-length test can range from 0 to 68. Butler et al. [7] found that a cutoff score of ≥ 9 produced acceptable sensitivity and specificity values.

**Table 1:** Descriptive statistics for the 17 items comprising the full-length COMM

**Item (“In the past 30 days…”)**	**Training dataset (*n *** **= 214)**		**Test dataset (*n *** **= 201)**	
	**Mean (SD)**	Median (IQR*)	**Mean (SD)**	Median (IQR*)
**1. How often have you had trouble with thinking clearly or had memory problems?**	1.3 (1.1)	1.0 (2.0)	1.5 (1.2)	1.0 (2.0)
**2. How often do people complain that you are not completing necessary tasks? (i.e., doing things that need to be done, such as going to class, work, or appointments)**	0.8 (1.1)	0.0 (1.0)	0.8 (1.0)	0.0 (1.0)
**3. How often have you had to go to someone other than your prescribing physician to get sufficient pain relief from your medications? (i.e. another doctor, the emergency room)**	0.3 (0.7)	0.0 (0.0)	0.2 (0.6)	0.0 (0.0)
**4. How often have you taken your medications differently from how they are prescribed?**	0.6 (0.9)	0.0 (1.0)	0.6 (0.9)	0.0 (1.0)
**5. How often have you seriously thought about hurting yourself?**	0.4 (0.8)	0.0 (1.0)	0.2 (0.6)	0.0 (0.0)
**6. How much of your time was spent thinking about opioid medications (having enough, taking them, dosing schedule, etc.)?**	1.0 (1.0)	1.0 (1.0)	0.8 (1.0)	1.0 (1.0)
**7. How often have you been in an argument?**	1.1 (0.9)	1.0 (1.0)	1.1 (1.0)	1.0 (2.0)
**8. How often have you had trouble controlling your anger (e.g., road rage, screaming, etc.)?**	0.7 (0.8)	1.0 (1.0)	0.8 (1.0)	0.0 (1.0)
**9. How often have you needed to take pain medications belonging to someone else?**	0.1 (0.4)	0.0 (0.0)	0.0 (0.2)	0.0 (0.0)
**10. How often have you been worried about how you’re handling your medications?**	0.5 (0.9)	0.0 (1.0)	0.4 (0.8)	0.0 (1.0)
**11. How often have others been worried about how you’re handling your medications?**	0.5 (0.9)	0.0 (1.0)	0.3 (0.7)	0.0 (0.0)
**12. How often have you had to make an emergency phone call or show up at the clinic without an appointment?**	0.2 (0.5)	0.0 (0.0)	0.1 (0.5)	0.0 (0.0)
**13. How often have you gotten angry with people?**	1.2 (0.8)	1.0 (1.0)	1.2 (1.0)	1.0 (2.0)
**14. How often have you had to take more of your medication than prescribed?**	0.6 (0.8)	0.0 (1.0)	0.5 (0.8)	0.0 (1.0)
**15. How often have you borrowed pain medication from someone else?**	0.1 (0.5)	0.0 (0.0)	0.0 (0.2)	0.0 (0.0)
**16. How often have you used your pain medicine for symptoms other than for pain (e.g. to help you sleep, improve your mood, or relieve stress)?**	0.2 (0.6)	0.0 (0.0)	0.2 (0.6)	0.0 (0.0)
**17. How often have you had to visit the emergency room?**	0.4 (0.7)	0.0 (1.0)	0.2 (0.5)	0.0 (0.0)

*IQR = Inter-quartile range.

In addition to taking the COMM, all participants were subjected to assessment via the ADBI, which is described in detail elsewhere [7,9]. As stated above, the ADBI is a combination measure that includes information from three sources. The first source of information is the Prescription Drug Use Questionnaire (PDUQ), a 42-item interview that evaluates pain condition, opioid use patterns, social and family factors, family history of pain and substance abuse syndromes, patient history of substance abuse, and psychiatric history [20]. To be consistent with Compton et al.’s finding that respondents scoring below 11 did not meet diagnostic criteria for a substance use disorder [20], a score ≥ 11 was defined as a positive result for the PDUQ in the current study. The ADBI’s second source of information is the Prescription Opioid Therapy Questionnaire (POTQ), an 11-item measure of opioid misuse that was adapted from the Physician Questionnaire of Aberrant Drug Behavior [6]. Each POTQ item is rated dichotomously (“yes” or “no”) by the patient’s treating physician; a positive answer to ≥ 2 items was taken to be a positive result for the POTQ. Finally, the ADBI’s third source of information is based on a urine toxicology screen. As in previous research [7], the current study defined a positive result as evidence of the subject having taken an illicit substance or another opioid medication that had not been prescribed. Once the information from each of the three sources had been collected, the results were pooled in order to obtain an overall classification. Specifically, the overall classification for the ADBI was defined as positive if (a) the result from the PDUQ was positive or (b) the results from both the POTQ and the urine toxicology screen were positive [9]. The ADBI was used in the current study to assess the concurrent validity of each method (curtailment, stochastic curtailment, and the full-length COMM); the sensitivity and specificity of these methods with respect to the ADBI will be reported in a later section.

### Curtailment and stochastic curtailment of the COMM

As mentioned previously, curtailment and stochastic curtailment rely on interim analysis of the respondent’s answers while assessment is in progress. Both of these methods thus require that the instrument be administered via computer. Use of computer-based testing to enhance the efficiency of questionnaires has been considered in numerous recent studies, due in part to the importance of computerized assessments in the Patient-Reported Outcomes Measurement Information System (PROMIS) project [21-23]. We next describe how curtailment and stochastic curtailment can be used in conjunction with computer-based testing to improve the efficiency of the COMM.

The fundamental idea of curtailment is to avoid administering items that have no bearing on the respondent’s final result from the questionnaire. By this logic, an assessment should be stopped if presenting further items will have no influence on the respondent’s final result. For example, suppose that a respondent is taking a computer-based version of the COMM, and that the cutoff value is ≥ 9 as suggested by Butler et al. [7]. Suppose further that the respondent’s scores to the first seven items are 1, 0, 2, 2, 1, 0, and 3, resulting in a cumulative score of 9 (the sum of the seven item scores). Because the respondent’s score has already reached the cutoff value, he/she is guaranteed to be “screened in” by the questionnaire (i.e., guaranteed to receive a positive result from the COMM), regardless of his/her responses to future items. A curtailment rule would observe this fact, halt testing after the seventh item, and prescribe that the respondent be “screened in,” thus eliminating the respondent burden that would have resulted from administering the final ten items. Conversely, consider a second respondent who has a cumulative score of 4 after taking the first 16 items. Regardless of the respondent’s answer to the seventeenth and final item (which has a maximum possible score of 4), he/she cannot possibly reach the cutoff value of 9. A curtailment rule would observe this fact and halt testing after the sixteenth item, prescribing that the respondent be “screened out” (i.e., that the respondent receive a negative result from the COMM). Finally, consider a third respondent who has a score of 0 after the first 15 items. Because this respondent cannot reach the cutoff value of 9—even if he/she receives a score of 4 on both the sixteenth item and seventeenth item—a curtailment rule would halt testing after the fifteenth item and prescribe that the respondent be “screened out.”

To summarize the above, a curtailment rule halts testing if (i) the respondent’s cumulative score reaches the cutoff value (in which case the respondent is “screened in”) or (ii) the respondent’s cumulative score is low enough that he/she cannot possibly reach the cutoff value, regardless of his/her future answers (in which case the respondent is “screened out”). The application of these rules to the COMM will be referred to as the COMM’s *curtailed version*. Because the curtailed version always prescribes the same result (“screened in” or “screened out”) as the full-length COMM, it also exhibits the same sensitivity and specificity as the full-length version. We note that administering the curtailed version is straightforward if the COMM is given by computer: the stopping rules of the curtailed version may be written as a simple look-up table and programmed into the computer for operational usage. An example of such a look-up table will be presented in a later section.

Stochastic curtailment is similar in concept to curtailment, but the former stops testing more liberally than the latter. In addition to stopping when it is *impossible* for future items to influence the final result of the questionnaire, stochastic curtailment also stops the test when future items are *unlikely* to influence the final result. Another (equivalent) way of describing stochastic curtailment is to state that the method stops testing when the probability of one of the possible outcomes (“screened in” or “screened out”) reaches or exceeds a certain threshold, given the respondent’s answers to the items administered thus far. Previous studies [18,19] recommended setting the threshold at 90%, 95%, or 99%.

To illustrate the above using a threshold of 95%, consider a respondent who is taking the COMM via computer and has completed the first five items of the questionnaire. Suppose that based on the respondent’s first five answers, his/her probability of receiving a “screened in” result from the full-length COMM is estimated to be at 89%; his/her probability of receiving a “screened out” result is estimated at 11%. Because both of these numbers are lower than 95%, stochastic curtailment continues the assessment by administering another item. Suppose next that based on the respondent’s answer to the sixth item, his/her estimated probability of receiving a “screened in” result rises to 97%; his/her probability of receiving a “screened out” result falls to only 3%. Because the 97% probability exceeds the 95% threshold for stopping, stochastic curtailment halts the questionnaire in favor of an immediate “screened in” result. If another respondent were to exhibit the reverse probabilities after six items (3% for “screened in” and 97% for “screened out”), he/she would be given an immediate “screened out” result. The application of such rules to the COMM will be referred to as the COMM’s *stochastically curtailed version*.

The most technical aspect of stochastic curtailment is how to determine the above probabilities at each *stage* of a given questionnaire (i.e., at each point of the questionnaire where an interim analysis is performed, which is typically after every item). Previous articles have explained the estimation of these probabilities in detail [18,19]. Briefly, the probabilities can be estimated and used effectively as long as a *training dataset* of prior respondents has been collected [18]. In other words, a prerequisite for stochastic curtailment to be used is that the complete questionnaire (here, the 17-item COMM) be administered to a group of initial respondents in a pilot study. The results of the pilot study are compiled as the training dataset, which is then used to estimate the probabilities in question. Specifically, the probabilities may be estimated by fitting a set of logistic regression models, one for each stage of the questionnaire, to the training dataset [19]. The logistic regression models are then used to estimate a respondent’s chance of receiving a “screened in” or “screened out” result, based on his/her cumulative score at any given stage. Specifics of the method can be found in Finkelman et al. [19], who also examined a nonparametric approach to probability estimation but found that logistic regression achieved a greater reduction in respondent burden.

We note that it is not necessary to perform any calculations of probabilities “in real time” as the respondent is taking the assessment. Instead, all calculations can be made in advance based on the data from the pilot study. In particular, the results of the logistic regression analyses can be used to determine which cumulative scores would result in early stopping at each stage of the questionnaire [19]. The stopping rules of stochastic curtailment may then be written in a simple look-up table that can be used for new respondents. An example of such a table will be presented in a later section; the table giving the stopping rules of stochastic curtailment will be compared to the analogous look-up table for curtailment.

Unlike curtailment, stochastic curtailment does not always produce the same result (“screened in” or “screened out”) as the full-length version of a given questionnaire. However, previous research found that when applied to the CES-D, the method achieves acceptable concordance with the full-length version of the instrument while substantially reducing the respondent burden [19]. The question of whether similar results can be obtained for the COMM will be addressed in a later section.

Although both curtailment and stochastic curtailment are simple in concept, they have been studied rigorously in the statistical literature. See Eisenberg and Ghosh [24] and Eisenberg and Simons [25] for information about the statistical properties of curtailment. See Davis and Hardy [26] and Lan et al. [27] for information about applications of stochastic curtailment to clinical trials, as well as theoretical results.

### Data analysis

The analysis involved data from the COMM’s initial validation study and cross-validation study, both of which were described in a previous section. Respondents who had not completed the full-length COMM (i.e., who were missing at least one of the 17 items) or had a missing ADBI classification were excluded from the investigation. The objective was to determine whether curtailment and stochastic curtailment could reduce the respondent burden of the COMM while maintaining the sensitivity and specificity of the assessment. To this end, a retrospective analysis was undertaken to determine the respondents’ test lengths and test outcomes (“screened in” or “screened out”) that would have occurred if curtailment or stochastic curtailment had been used. Such *post-hoc simulation* is common when studying the efficiency of questionnaires [14,18,19,21].

As explained previously, stochastic curtailment requires the analysis of training data from an initial set of respondents before the method can be used operationally. To mimic the sequence of events as would occur in practice, the logistic regression models utilized in stochastic curtailment were first fitted to the dataset from the COMM’s initial validation study. This dataset was thus defined as the “training dataset” for the purpose of the investigation. The results of the logistic regression analysis were then used to find the stopping rules of stochastic curtailment (i.e., to find the specific cumulative COMM scores that would result in early stopping at each stage of the test, and write them in look-up tables). Three different probability thresholds—90%, 95%, and 99%–were examined, resulting in three separate sets of stopping rules. These rules will hereafter be referred to as SC-90, SC-95, and SC-99, respectively (“SC” standing for “stochastic curtailment”). Evaluation of the different stochastically curtailed versions was then performed on the cross-validation dataset; that is, the test length and test outcome that would have resulted from the SC-90, SC-95, and SC-99 stopping rules were determined for each respondent in the cross-validation dataset (hereafter referred to as the “test dataset”). The performance of the full-length COMM and its curtailed version were also evaluated using the same test dataset so that a standardized comparison could be made. In particular, for each version of the questionnaire, the following statistics were computed: sensitivity and specificity with respect to the full-length COMM, sensitivity and specificity with respect to the ADBI, average number of items administered, standard deviation of the number of items administered, and percentage of respondents whose tests stopped early (i.e., prior to the seventeenth item). The last three of these measures relate to respondent burden. Descriptive statistics for each item (mean, standard deviation, median, and inter-quartile range) were also calculated. All analyses were carried out using the statistical software package R (Version 2.11.1). We note that the partitioning of the data into separate training and test datasets, as was done in our analysis, is recommended in the statistical literature in lieu of using the same dataset for both model fitting and performance evaluation; the latter approach can result in misleadingly positive results [28].

The Institutional Review Board at Tufts Medical Center and Tufts University Health Sciences Campus granted exempt status for this research project.

## Results

Following the application of exclusion rules, the training dataset had a final sample size of *n* = 214, while the test dataset had a final sample size of *n* = 201. In the training dataset, the mean (SD) COMM score was 10.1 (7.5); 104 respondents (48.6%) were “screened in” by the COMM using a ≥ 9 cutoff, and 73 respondents (34.1%) were classified as positive by the ADBI. In the test dataset, the mean (SD) COMM score was 8.9 (6.9); 86 respondents (42.8%) were “screened in” by the COMM using a ≥ 9 cutoff, and 64 respondents (31.8%) were classified as positive by the ADBI.

Table 1 presents descriptive statistics for each item of the COMM. In both the training and test datasets, the median value for every item was either 0 (“never”) or 1 (“seldom”). For 16 of the 17 items, the median in the training dataset was equal to the median in the test dataset; the lone exception was item 8 (“How often have you had trouble controlling your anger (e.g., road rage, screaming, etc.)?”), which had a median of 1 in the training dataset and a median of 0 in the test dataset. In both datasets, the item with the highest mean was item 1 (“How often have you had trouble with thinking clearly or had memory problems?”), which had a mean of 1.3 in the training dataset and 1.5 in the test dataset. No item’s mean in the training dataset was more than 0.2 from its mean in the test dataset.

Table 2 presents stopping boundaries for each of the sequential methods under study (curtailment, SC-99, SC-95, and SC-90). That is, for each sequential method examined herein, the table indicates the scores that result in early stopping for each stage of the test. Scores that produce a “screened out” result are labeled “Negative stopping,” while scores that produce a “screened in” result are labeled “Positive stopping.” For example, after 10 items have been completed, curtailment never stops to produce a “screened out” result (as denoted “N/A” in Table 2 to indicate “Not Applicable”), but it stops to produce a “screened in” result if the respondent’s score is ≥ 9 at that stage. Continuing in the same row of the table, SC-99 stops after 10 items if the respondent’s score is ≤ 2 (“screened out”) or ≥ 9 (“screened in”); SC-95 stops if the respondent’s score is ≤ 3 (“screened out”) or ≥ 8 (“screened in”); SC-90 stops if the respondent’s score is ≤ 4 (“screened out”) or ≥ 8 (“screened in”). These results illustrate that SC-90 is the most liberal stopping rule of the four (it has the largest range of scores that result in early stopping), followed by SC-95, SC-99, and curtailment (the most conservative stopping rule).

**Table 2:** **Stopping boundaries for curtailment and stochastic curtailment (based on the training dataset: *n *** **= 214)**

	**Curtailment**		**SC-99**		**SC-95**		**SC-90**	
**Items completed**	**Negative stopping**	**Positive stopping**	**Negative stopping**	**Positive stopping**	**Negative stopping**	**Positive stopping**	**Negative stopping**	**Positive stopping**
**1**	N/A	N/A	N/A	N/A	N/A	N/A	N/A	Score = 4
**2**	N/A	N/A	N/A	Score = 8	N/A	Score ≥ 6	N/A	Score ≥ 5
**3**	N/A	Score ≥ 9	N/A	Score ≥ 9	N/A	Score ≥ 7	N/A	Score ≥ 6
**4**	N/A	Score ≥ 9	N/A	Score ≥ 8*	N/A	Score ≥ 7	Score = 0	Score ≥ 6
**5**	N/A	Score ≥ 9	N/A	Score ≥ 8*	N/A	Score ≥ 7	Score = 0	Score ≥ 6
**6**	N/A	Score ≥ 9	N/A	Score ≥ 9	Score = 0	Score ≥ 8	Score ≤ 1	Score ≥ 7
**7**	N/A	Score ≥ 9	Score = 0	Score ≥ 9	Score ≤ 2	Score ≥ 8	Score ≤ 2	Score ≥ 7
**8**	N/A	Score ≥ 9	Score ≤ 1	Score ≥ 9	Score ≤ 3	Score ≥ 8	Score ≤ 3	Score ≥ 8
**9**	N/A	Score ≥ 9	Score ≤ 1	Score ≥ 9	Score ≤ 3	Score ≥ 8	Score ≤ 3	Score ≥ 8
**10**	N/A	Score ≥ 9	Score ≤ 2	Score ≥ 9	Score ≤ 3	Score ≥ 8	Score ≤ 4	Score ≥ 8
**11**	N/A	Score ≥ 9	Score ≤ 3	Score ≥ 9	Score ≤ 4	Score ≥ 9	Score ≤ 4	Score ≥ 8
**12**	N/A	Score ≥ 9	Score ≤ 3	Score ≥ 9	Score ≤ 4	Score ≥ 9	Score ≤ 4	Score ≥ 8
**13**	N/A	Score ≥ 9	Score ≤ 4	Score ≥ 9	Score ≤ 5	Score ≥ 9	Score ≤ 6	Score ≥ 9
**14**	N/A	Score ≥ 9	Score ≤ 5	Score ≥ 9	Score ≤ 6	Score ≥ 9	Score ≤ 6	Score ≥ 9
**15**	Score = 0	Score ≥ 9	Score ≤ 5	Score ≥ 9	Score ≤ 6	Score ≥ 9	Score ≤ 7	Score ≥ 9
**16**	Score ≤ 4	Score ≥ 9	Score ≤ 6	Score ≥ 9	Score ≤ 6	Score ≥ 9	Score ≤ 7	Score ≥ 9
**17**	Score ≤ 8	Score ≥ 9	Score ≤ 8	Score ≥ 9	Score ≤ 8	Score ≥ 9	Score ≤ 8	Score ≥ 9

*In the constrained version of stochastic curtailment, this stopping boundary is ≥ 9.

Most of the stopping boundaries in Table 2 are *monotonically nondecreasing:* as the stage of the test advances, the cutoff score required for early stopping generally does not decrease. The one exception is SC-99, which has a “Positive stopping” requirement of ≥ 9 at stage 3 but only a requirement of ≥ 8 at stages 4 and 5. This result may be surprising, as a score of 8 would intuitively be considered less evidence of a final “screened in” result at stage 4 or 5 than at stage 3. The result is possible, however, because the stopping boundaries were obtained by fitting a separate logistic regression model at each stage of the questionnaire, without constraining these boundaries to be monotonic. If monotonic boundaries are preferred, a simple *constrained version* of stochastic curtailment may be defined whereby the boundaries are adjusted to be nondecreasing. To be conservative, the stopping rule at stages 4 and 5 would be adjusted upward to ≥ 9, as opposed to adjusting the stopping rule at stage 3 downward to ≥ 8. The constrained boundaries are noted in Table 2.

Table 3 presents the sensitivity and specificity values of each method, as well as statistics related to respondent burden. The full-length COMM had a sensitivity of 0.703 and a specificity of 0.701 for predicting the ADBI. Curtailment and SC-99 always produced the same result (“screened in” or “screened out”) as the full-length COMM; hence, these methods had a sensitivity and specificity of 1 for predicting the full-length COMM, as well as a sensitivity and specificity of 0.703 and 0.701, respectively, for predicting the ADBI. SC-95 and SC-90 did not always match the result of the full-length COMM, but their sensitivities for it were 0.977 and 0.965, respectively; their specificities for it were 1 and 0.991, respectively. Moreover, these methods exhibited sensitivities of 0.688 or more, and specificities of 0.708 or more, for predicting the ADBI.

**Table 3:** **Sensitivity, specificity, and respondent burden of each method (based on the test dataset: *n *** **= 201)**

	**Predicting the full-length COMM**		**Predicting the ADBI**		**Average test length**	**SD of test length**	**% of test lengths < 17**
	**Sensitivity**	**Specificity**	**Sensitivity**	**Specificity**			
**Full-length COMM**	1	1	0.703	0.701	17.0	0.0	0.0
**Curtailment**	1	1	0.703	0.701	13.3	4.2	71.6
**SC-99**	1	1	0.703	0.701	10.7	3.9*	88.1
**SC-95**	0.977	1	0.703	0.715	8.7	4.0	90.0
**SC-90**	0.965	0.991	0.688	0.708	7.0	4.2	96.5

*In the constrained version of stochastic curtailment, the SD of test length was 3.8 for SC-99.

Regarding respondent burden, the full-length COMM, by definition, never stops prior to the seventeenth item; therefore, its average (SD) test length was 17.0 (0.0), and its percentage of respondents whose tests stopped early was 0%. The average (SD) test lengths for curtailment, SC-99, SC-95, and SC-90 were 13.3 (4.2), 10.7 (3.9), 8.7 (4.0), and 7.0 (4.2), respectively. The percentage of respondents whose tests stopped early was at least 71.6% for every sequential stopping method; the highest such value was 96.5%, which was observed for SC-90.

We note that if the constrained version of SC-99 were used, the results would be nearly identical to those of SC-99. The two methods had the same sensitivity values, specificity values, and percentages of respondents whose tests stopped early. Their average test lengths were equal to one decimal place. The standard deviation of test lengths was 3.8 for the constrained SC-99, as opposed to 3.9 for SC-99.

## Discussion

There are many well-known benefits of computer-based testing, including automated scoring, immediate data entry, and facilitated tracking of change over time [29]. As described above, computer-based testing can also be coupled with sequential stopping rules to reduce the respondent burden of an assessment. Two such stopping rules, both of which enhanced the efficiency of the Medicare Health Outcomes Survey [18] and the CES-D [19] in previous post-hoc simulations, are curtailment and stochastic curtailment. However, prior to the current study, neither of these stopping rules had been investigated for use with the COMM.

Results of the study indicated that both curtailment and stochastic curtailment have the potential to reduce the respondent burden of the COMM without compromising sensitivity and specificity. Curtailment lowered the average test length by 22% while maintaining the same sensitivity and specificity as the full-length COMM. SC-99 also maintained sensitivity and specificity values identical to those of the full-length COMM while reducing the average test length by 37%. The sensitivities and specificities of SC-95 and SC-90 for predicting the ADBI were within 1.5% of those of the full-length COMM, while these methods reduced the average test length by 49% and 59%, respectively.

Which sequential stopping rule to use operationally will depend on the practitioner’s desired level of concordance with the full-length COMM. If the practitioner requires that the result of the shortened version (“screened in” or “screened out”) match that of the full-length version for all respondents, then curtailment is the correct method to use. We note that the result of SC-99 also matched that of the full-length COMM for every respondent considered in the current study; however, due to its stochastic nature (and unlike curtailment), SC-99 is not guaranteed to match the full-length COMM for 100% of future respondents. If the practitioner is willing to accept a possible decrement in sensitivity and/or specificity to achieve a greater reduction in respondent burden, then the more aggressive SC-99 may be preferred to curtailment. Further gains in average test length can be achieved by using the more liberal SC-95 or SC-90, although these methods may also exhibit less concordance with the result of the full-length COMM.

While the methods examined herein produced substantial improvements in efficiency, these improvements could potentially be enhanced by considering the *ordering* of the COMM items. The current article assumed that items would be presented in the same order that they are given in the paper-and-pencil version of the COMM. This assumption was made to promote comparability between the computerized and paper-and-pencil versions. However, it ignores the possibility that in a computerized version of the COMM, items could be judiciously ordered to augment the gains made by curtailment and stochastic curtailment. Previous research [30] has shown that by presenting the most informative items (e.g., the items selected first by a stepwise logistic regression procedure) at the beginning of the test, the average test lengths of curtailed and stochastically curtailed assessments are reduced without loss of classification accuracy. Future research should investigate the impact of item ordering in a computer-based COMM.

Another mechanism by which the COMM’s statistical properties could potentially be improved is the use of a more sophisticated classification model. The simple ≥ 9 cutoff rule is desirable from the standpoints of interpretability and logistical ease, but more rigorous statistical classification tools might achieve better sensitivity and specificity. Curtailment and stochastic curtailment have previously been studied alongside a multiple logistic regression classification rule [18,30]; such a rule has the added benefit of facilitating the inclusion of demographic information in the model if desired. Additionally, the classification of respondents via Item Response Theory (IRT) and computerized adaptive testing (CAT), which would allow the item ordering to be individualized at the respondent level, could be explored. Although the suitability of IRT and CAT for application to the COMM has not yet been examined, a comparison between the curtailed, stochastically curtailed, and CAT-based versions of the COMM could be illuminating. Previous comparisons using the CES-D suggested that stochastic curtailment can achieve reductions in test length similar to those of CAT while exhibiting greater concordance with the classifications of the full-length instrument [19].

Regarding limitations of the current study, one was its retrospective nature: responses were analyzed post-hoc rather than collecting data prospectively. However, such post-hoc simulation to establish a “proof-of-concept” is a typical first step in evaluating potential computer-based testing procedures [14,18,19,21]. A second limitation was the sample size of the study, which was smaller than the sample sizes of previous applications of curtailment and stochastic curtailment [18,19]. However, the fact that the stopping rules were successful when applied to the test dataset, despite having been trained on a relatively modest-sized training dataset, suggests the robustness of the methodology. Third, the methods were evaluated using only one test dataset, limiting the generalizability of the results. We further caution readers that while the look-up table for curtailment (Table 2) is applicable to any population for which a ≥ 9 cutoff is appropriate, the look-up tables for SC-99, SC-95, and SC-90 (also shown in Table 2) are sample-specific and may not be suitable for other populations. Therefore, before stochastic curtailment is used for a different population of respondents, new look-up tables should be calculated based on pilot data from that population. See Finkelman et al. [19] for a thorough description of how to calculate such look-up tables.

It should be reiterated that at 17 items, the full-length COMM is not unduly time-consuming for many individuals who take it. Nevertheless, reducing the respondent burden of an assessment can result in benefits such as an enhanced response rate [11], including among members of compromised subgroups [10]. Other potential advantages of shortening an instrument are an improvement in the quality of answers obtained [12], a reduction in respondents’ stress levels [13], and a lower drop-out rate [13]. Alleviating the respondent burden may be particularly important for the COMM, given that this questionnaire was designed to be administered on multiple occasions to track patient status over time [7].

We also emphasize that the COMM was not designed as a standalone mechanism for classification. Rather, it was developed as a screening tool to help clinicians in their assessment of risk for opioid misuse [7,9]. Therefore, the curtailed and stochastically curtailed versions should also be regarded as aids to clinicians, rather than as standalone classification tools.

Further investigations should be conducted to replicate this study’s results in different populations. In addition to retrospective analyses, curtailment and stochastic curtailment should be pilot-tested in live computer-based administrations. Subjects from compromised subpopulations, such as those with physical ailments and those who are at a low reading level, should be included in the pilot testing, considering that these subpopulations are most likely to benefit from reduced test lengths [10]. The results of the live tests should be compared to the results of post-hoc simulation. All of these steps will be undertaken in future work.

## Conclusions

Within the limitations of the study, results suggest that curtailment and stochastic curtailment reduce the respondent burden of the COMM without compromising its sensitivity and specificity. Therefore, these sequential methods have the potential to improve the COMM’s response rate and enhance the quality of its respondents’ answers, particularly among members of compromised subpopulations. Curtailment and stochastic curtailment should be considered for future computer-based administrations of the COMM.

## Abbreviations

ADBI: Aberrant drug behavior index; CES-D: Center for epidemiologic studies-depression scale; COMM: Current opioid misuse measure; IQR: Inter-quartile range; N/A: Not applicable; PDUQ: Prescription drug use questionnaire; POTQ: Prescription opioid therapy questionnaire; PROMIS: Patient-reported outcomes measurement information system; SC-90: Stochastic curtailment using a 90% probability threshold for early stopping; SC-95: Stochastic curtailment using a 95% probability threshold for early stopping; SC-99: Stochastic curtailment using a 99% probability threshold for early stopping; SD: Standard deviation.

## Competing interests

SFB is an employee and shareholder of Inflexxion, Inc. Inflexxion holds the copyright for the Current Opioid Misuse Measure (COMM)®.

## Authors’ contributions

MDF contributed to the conception of the study, analyzed the data, and prepared the manuscript. RJK contributed to the conception of the study and made significant comments on the manuscript. DZ and NS made significant comments on the interpretation of data and the manuscript. SFB contributed to the conception of the study, acquired the data, and made significant comments on the manuscript. All authors read and approved the final manuscript.

## Authors’ information

MDF is an assistant professor, RJK is a professor, and DZ is an associate professor, all at Tufts University School of Dental Medicine. NS is an assistant professor at VU University Amsterdam. SFB is senior vice president and Chief Science Officer at Inflexxion, Inc.

## Pre-publication history

The pre-publication history for this paper can be accessed here:

http://www.biomedcentral.com/1471-2288/13/126/prepub
